# Supplementary material for: Training Recurrent Neural Networks for BrdU Detection with Oxford Nanopore Sequencing: Guidance and Lessons Learned
Source: Genes (Basel). 2025 Nov 10;16(11):1356. doi: 10.3390/genes16111356 (PMC12652529; doi:10.3390/genes16111356)
Supplement: Supplementary file 1 [file genes-16-01356-s001.zip › File S1. Step-by-step tutorial for data preparation and model trainingpdf.pdf]

Data preparation for training BiGRU RNNs to detect BrdU with Nanopore DNA sequencing

Haibo Liu

2025-08-14

Contents

Preparing data for basecalling raw Nanopore sequencing data with Dorado 1
Basecalling with Dorado 3
Converting BAM to fastq using SAMtools 4
Generating minimap2 index for alignment 4
Aligning reads to the reference genome using minimap2 5
Extracting valid fastq with mapping quality of 60 5
Converting multi-read fast5, pod5 or slow5/blow5 to single-read fast5 6
Adding read sequence information from the fastq file to individual single- 6
Repacking fast5 7
Resquiggling using Tombo 8
Filtering resquiggled single-read fast5 files to remove fast5 files without 8
Extracting event-specific statistics from the resquiggled single-read 9

Preparing data for basecalling raw Nanopore sequencing data with Dorado

This step depends on the starting format of your Nanopore DNA sequencing data, which can be single-read fast5, multi-read fast5, pod5, slow5 or blow5. Formats other than single-read fast5 can hold one or more reads. Since Dorado only takes data in the pod5 format as input, if you start with aother formats you will have to convert it to pod5. All the processing steps were done in a high performance computer cluster with Nvidia GPU installed, with the LSF (Load Sharing Facility) system for workload management and job scheduling. When possible, singularity images will be used, otherwise conda virtual environments or system-installed modules will be used.

Convert single-read fast5 to multi-read fast5 using ont-fast5-api

First download ont-fast5-api (https://github.com/nanoporetech/ont_fast5_api) singularity image from Biocontainer (https://biocontainers.pro/tools/ont-fast5-api) by run the following command:

```
mkdir -p docs scripts data logs

singularity run https://depot.galaxyproject.org/singularity/ont-fast5-api:4.1.3--pyhdfd78af_0
mv ont-fast5-api:4.1.3--pyhdfd78af_0 scripts
```

Then use the image to convert single-read fast5 files to multi-read fast5 files by running the following job script.

```
#!/bin/bash

#BSUB -n 8  # minmal numbers of processors required for a parallel job
#BSUB -R rusage[mem=8000] # ask for memory 5G
```

```
#BSUB -W 4:00 #limit the job to be finished in 12 hours
#BSUB -J "fastQC[1-2]"
#BSUB -q long   # which queue we want to run in
#BSUB -o logs/out.%J.%I.txt # log
#BSUB -e logs/err.%J.%I.txt # error
#BSUB -R "span[hosts=1]" # All hosts on the same chassis"
##BSUB -w "done(33544)"

i=$(($LSB_JOBINDEX- 1))
mkdir -p logs

# Each directory of 'single_fast5_dir' contains many single-read fast5 files
single_fast5_dir=(`ls data/000.single.fast5/* -d`)
outdir=(`ls data/000.single.fast5/* -d | \
         perl -p -e 's{data/000.single.fast5/(.+)}{$1}'`)
save_path=data/000.multi_fast5/${outdir[$i]}
mkdir -p $save_path

singularity run scripts/ont-fast5-api:4.1.3--pyhdfd78af_0  \
        single_to_multi_fast5 --input_path ${single_fast5_dir[$i]} \
        --save_path $save_path  \
        --threads 8  \
        --recursive
```

Convert multi-read fast5 to pod5 using pod5

pod5 has been installed in the HPC as module. It can be downloaded from https://github.com/nanoporetech/pod5-file-format. Or more conveniently, download a singularity image for it from Biocontainer as follows:

```
singularity run https://depot.galaxyproject.org/singularity/pod5:0.3.15--pyhdfd78af_0
mv pod5:0.3.15--pyhdfd78af_0 scripts
```

Generate a file containing the full paths to each multi-read fast5 files and the unique names to be used for naming pod5 files.

```
find data/000.multi_fast5 -name '*.fast5' | \
       perl -p -e 's{(vector/000.multi_fast5/(.+?)/(.+).fast5)}{$1\t$2_$3}' \
       > docs/yeast.train.test.list
```

Convert multi-read fast5 to pod5 by running the following job script.

```
#!/bin/bash

#BSUB -n 1  # minmal numbers of processors required for a parallel job
#BSUB -R rusage[mem=4000] # ask for memory 5G
#BSUB -W 4:00 #limit the job to be finished in 12 hours
#BSUB -J "fastQC[1-32]"
#BSUB -q short   # which queue we want to run in
#BSUB -o logs/out.%J.%I.txt # log
#BSUB -e logs/err.%J.%I.txt # error
#BSUB -R "span[hosts=1]" # All hosts on the same chassis"
##BSUB -w "done(33544)"

i=$(($LSB_JOBINDEX- 1))
mkdir -p logs
```

```
module load pod5/0.3.15
list=docs/yeast.train.test.list
fast5=(`cut -f1 $list`)
name=(`cut -f2  $list`)
mkdir -p data/002.pod5

pod5 convert fast5 ${fast5[$i]}  --output data/002.pod5/${name[$i]}.pod5

# using the singularity image

# singularity run  scripts/pod5:0.3.15--pyhdfd78af_0  \
#      pod5 convert fast5 ${fast5[$i]}  --output data/002.pod5/${name[$i]}.pod5
```

Convert slow5/blow5 to pod5

slow5 is a new file format for storing signal data from Oxford Nanopore Technologies (ONT) devices, which is more efficient. In this study, we don't have Nanopore data in the format of slow5/blow5. If you encounter such type of data, you can convert slow5 into single-read fast5 using **slow5tools** (https://github.com/hasindu2008/slow5tools), by reference to the documentation of the slow5tools (https://hasindu2008.github.io/slow5tools/commands.html). Then convert single-read fast5 to pod5 as described at steps 1.1 to 1.3.

Basecalling with Dorado

Dorado is the latest basecaller for Nanopore data developed by the Oxford Nanopore Technologies. It can be downloaded from https://github.com/nanoporetech/dorado. Here, we used the Dorado installed in the HPC as module. Before running Dorado, a basecalling model for data generated with the R9.4.1 chemistry has to been downloaded. We use the super accurate model, dna_r9.4.1_e8_sup@v3.6.

```
module load dorado/0.9.1
dorado download --model dna_r9.4.1_e8_sup@v3.6 --models-directory docs
```

```
#!/bin/bash

#BSUB -n 8  # minmal numbers of processors required for a parallel job
#BSUB -R rusage[mem=1000] # ask for memory 5G
#BSUB -W 4:00 #limit the job to be finished in 12 hours
#BSUB -J "fastQC[1-32]"
#BSUB -q  gpu   # which queue we want to run in
#BSUB -o logs/out.%J.%I.txt # log
#BSUB -e logs/err.%J.%I.txt # error
#BSUB -R "span[hosts=1]" # All hosts on the same chassis"
##BSUB -w "done(33544)"

i=$(($LSB_JOBINDEX- 1))
mkdir -p logs

module load dorado/0.9.1

pod5=(`ls  data/002.pod5/*.pod5`)
name=(`ls  data/002.pod5/*.pod5 | perl -p -e  's{.+/(.+?).pod5}{$1}' `)
out=data/003.dorado_basecalled/
mkdir -p $out
```

```
dorado  basecaller --no-trim \
       -x cuda:0 \
       docs/dna_r9.4.1_e8_sup@v3.6  ${pod5[$i]}  > $out/${name[$i]}_dorado.bam
```

Converting BAM to fastq using SAMtools

```
#!/bin/bash

#BSUB -n 8  # minmal numbers of processors required for a parallel job
#BSUB -R rusage[mem=8000] # ask for memory 5G
#BSUB -W 24:00 #limit the job to be finished in 12 hours
#BSUB -J "fastQC[1-32]"
#BSUB -q long  # which queue we want to run in
#BSUB -o logs/out.%J.%I.txt # log
#BSUB -e logs/err.%J.%I.txt # error
#BSUB -R "span[hosts=1]" # All hosts on the same chassis"
##BSUB -w "done(195835)"

i=$(($LSB_JOBINDEX- 1))
mkdir -p logs

module load samtools/1.16.1

out=data/004.dorado_basecalled_fastq
bam=(`ls $out/*.bam`)
name=(`ls $out/*.bam | perl -p -e 's{.+/(.+?).bam}{$1}'`)
mkdir -p $out

set -euo pipefail
samtools fastq -T "*" ${bam[$i]} | gzip -9 > $out/${name[$i]}.fastq.gz
```

Generating minimap2 index for alignment

Download the reference genome from Ensembl.

```
#!/bin/bash

#BSUB -n 8  # minmal numbers of processors required for a parallel job
#BSUB -R rusage[mem=8000] # ask for memory 5G
#BSUB -W 24:00 #limit the job to be finished in 12 hours
#BSUB -J "fastQC[1]"
#BSUB -q long  # which queue we want to run in
#BSUB -o logs/out.%J.%I.txt # log
#BSUB -e logs/err.%J.%I.txt # error
#BSUB -R "span[hosts=1]" # All hosts on the same chassis"
##BSUB -w "done(195835)"

i=$(($LSB_JOBINDEX- 1))
mkdir -p logs

module load minimap2/2.26

set -euo pipefail
```

```
wget https://ftp.ensembl.org/pub/release-114/fasta/saccharomyces_cerevisiae/dna/Saccharomyces_cerevisia

gunzip docs/Saccharomyces_cerevisiae.R64-1-1.dna.toplevel.fa.gz
ref_fasta=docs/Saccharomyces_cerevisiae.R64-1-1.dna.toplevel.fa
index=docs/yeast.mmi

minimap2 -x map-ont -d $index  ${ref_fasta}
```

Aligning reads to the reference genome using minimap2

Download the reference genome from Ensembl.

```
#!/bin/bash

#BSUB -n 8  # minmal numbers of processors required for a parallel job
#BSUB -R rusage[mem=8000] # ask for memory 5G
#BSUB -W 24:00 #limit the job to be finished in 12 hours
#BSUB -J "fastQC[1-32]"
#BSUB -q long  # which queue we want to run in
#BSUB -o logs/out.%J.%I.txt # log
#BSUB -e logs/err.%J.%I.txt # error
#BSUB -R "span[hosts=1]" # All hosts on the same chassis"
##BSUB -w "done(195835)"

i=$(($LSB_JOBINDEX- 1))
mkdir -p logs

module load minimap2/2.26
set -euo pipefail

in_dir=data/004.dorado.basecalled.fastq
fastq=(`ls ${in_dir}/*.fastq.gz`)
name=(`ls ${in_dir}/*.fastq.gz | perl -p -e 's{.+/(.+?).fastq.gz)}{$1}'`)
index=docs/yeast.mmi
out=data/005.filtered.bam

minimap2 -ax map-ont -y -t 8 -B 4 -O 4,24  --sam-hit-only \
    $index  $fastq | samtools view -bh  -q 60 - > $out/${name[$i]}.mapq60.bam
```

Extracting valid fastq with mapping quality of 60

```
#!/bin/bash

#BSUB -n 1  # minmal numbers of processors required for a parallel job
#BSUB -R rusage[mem=2000] # ask for memory 5G
#BSUB -W 24:00 #limit the job to be finished in 12 hours
#BSUB -J "fastQC[1-344]%50"
#BSUB -q long  # which queue we want to run in
#BSUB -o logs/out.%J.%I.txt # log
#BSUB -e logs/err.%J.%I.txt # error
#BSUB -R "span[hosts=1]" # All hosts on the same chassis"
##BSUB -w "done(195835)"
```

```
i=$(($LSB_JOBINDEX- 1))
mkdir -p logs

module load samtools/1.16.1
module load seqtk

bam=(`ls data/005.filtered.bam/*.bam`)
fastq=(`data/004.dorado.basecalled.fastq/*.fastq`)
name=(`ls data/005.filtered.bam/*.bam | perl -p -e 's{.+/(.+?).mapq60.bam}{$1}'`)
out=data/004.filtered.fastq

mkdir -p $out

awk 'BEGIN{FS=OFS="\t"} !/^@/ {print $1}' <(samtools view ${bam[$i]}) | \
     sort -u | seqtk subseq ${fastq[$i]}  -  > $out/${name[$i]}.fastq
```

Converting multi-read fast5, pod5 or slow5/blow5 to single-read fast5

This step is only **NEEDED** if the raw Nanopore sequencing data is not in the single-read fast5 format. Here only provide the script for converting multi-read fast5 to single-read fast5. If starting with pod5, use **pod5 convert to_fast5** command. If starting with slow5/blow5, use **slow5tools** (https://github.com/hasindu2008/slow5tools), by reference to the documentation of the slow5tools (https://hasindu2008.github.io/slow5tools/commands.html).

```
#!/bin/bash

#BSUB -n 8  # minmal numbers of processors required for a parallel job
#BSUB -R rusage[mem=1000] # ask for memory 5G
#BSUB -W 4:00 #limit the job to be finished in 12 hours
#BSUB -J "fastQC[1-2]%50"
#BSUB -q short   # which queue we want to run in
#BSUB -o logs/out.%J.%I.txt # log
#BSUB -e logs/err.%J.%I.txt # error
#BSUB -R "span[hosts=1]" # All hosts on the same chassis"
##BSUB -w "done(33544)"

i=$(($LSB_JOBINDEX- 1))
mkdir -p logs

multi_fast5=(`ls data/*.fast5`)
out_dir=(`ls data/*.fast5 | perl -p -e 's{.+/(.+?).fast5}{$1}'`)
save_path=data/000.single_fast5/${out_dir[$i]}
mkdir -p $save_path

singularity run  scripts/ont-fast5-api:4.1.3--pyhdfd78af_0  \
        multi_to_single_fast5 --input_path  ${multi_fast5[$i]} \
        --save_path  $save_path   \
        --recursive
```

Adding read sequence information from the fastq file to individual single-

read fast5 using Tombo

A singularity image for Tombo is downloaed from the BioContainer registry as follows:

```
singularity run https://depot.galaxyproject.org/singularity/ont-tombo:1.5.1--py37r36h70f9b12_2
```

Qualified read sequence information from the fastq files is added to corresponding single-read fast5 files using Tombo to get basecalled single-read fast5 files.

```
#!/bin/bash

#BSUB -n 8  # minmal numbers of processors required for a parallel job
#BSUB -R rusage[mem=1000] # ask for memory 5G
#BSUB -W 4:00 #limit the job to be finished in 12 hours
#BSUB -J "fastQC[2]%50"
#BSUB -q short   # which queue we want to run in
#BSUB -o logs/out.%J.%I.txt # log
#BSUB -e logs/err.%J.%I.txt # error
#BSUB -R "span[hosts=1]" # All hosts on the same chassis"
##BSUB -w "done(33544)"

i=$(($LSB_JOBINDEX- 1))
mkdir -p logs

single_fast5=(`ls data/000.single_fast5/* -d`)
fastq=(`ls data/004.filtered.fastq/*.fastq`)

singularity run  scripts/ont-tombo:1.5.1--py37r36h70f9b12_2  \
  tombo preprocess annotate_raw_with_fastqs \
  --fast5-basedir ${single_fast5[$i]} \
  --fastq-filenames ${fastq[$i]} --overwrite
```

Repacking fast5

This step is needed only if some group(s) of the fast5 files have been deleted using the h5delete tool. Otherwise, subsequent resquiggline will fail.

```
#!/bin/bash

#BSUB -n 1  # minmal numbers of processors required for a parallel job
#BSUB -R rusage[mem=1000] # ask for memory 5G
#BSUB -W 2:00 #limit the job to be finished in 12 hours
#BSUB -J "fastQC[2-6843]%50"
#BSUB -q short   # which queue we want to run in
#BSUB -o logs/out.%J.%I.txt # log
#BSUB -e logs/err.%J.%I.txt # error
#BSUB -R "span[hosts=1]" # All hosts on the same chassis"
##BSUB -w "done(33544)"

i=$(($LSB_JOBINDEX- 1))
mkdir -p logs

module load hdf5

single_fast5=(`ls  data/000.single_fast5/*/*.fast5`)
in_dir=(`ls  data/000.single_fast5/*/*.fast5 | perl -p -e 's{(.+)/.+}{$1}'`)
name=(`ls  data/000.single_fast5/*/*.fast5 | perl -p -e 's{.+/(.+)}{$1}'`)
```

```
out=(`ls data/000.single_fast5/*/*.fast5 | perl -p -e 's{ data/000.single_fast5/(.+?)/.+?.fast5}{data/0

mkdir -p ${out[$i]}

h5repack -f GZIP=5 ${single_fast5[$i]}   ${out[$i]}/${name[$i]}
```

Resquiggling using Tombo

```
#!/bin/bash

#BSUB -n 12  # minmal numbers of processors required for a parallel job
#BSUB -R rusage[mem=8000] # ask for memory 5G
#BSUB -W 72:00 #limit the job to be finished in 12 hours
#BSUB -J "fastQC[1-2]%50"
#BSUB -q long   # which queue we want to run in
#BSUB -o logs/out.%J.%I.txt # log
#BSUB -e logs/err.%J.%I.txt # error
#BSUB -R "span[hosts=1]" # All hosts on the same chassis"
##BSUB -w "done(33544)"

i=$(($LSB_JOBINDEX- 1))
mkdir -p logs

single_fast5=(`ls data/001.repacked.single.fast5/barcode0* -d`)
ref_fasta=docs/Saccharomyces_cerevisiae.R64-1-1.dna.toplevel.fa

singularity run  scripts/ont-tombo:1.5.1--py37r36h70f9b12_2  \
  tombo resquiggle --dna \
  --processes 12 \
  --ignore-read-locks \
  --overwrite \
  --threads-per-process 4 \
  --sequence-length-range  500 5000000 \
  --signal-length-range 500 500000000 \
  --include-event-stdev \
  --basecall-group Basecall_1D_000 \
  --num-most-common-errors 5 \
  ${single_fast5[$i]}  ${ref_fasta}
```

Filtering resquiggled single-read fast5 files to remove fast5 files without

events group

Save the following R scripts as “scripts/filter.resquiggle.fast5.R”. To run the scripts, the R base and rhdf5 package need to be installed.

```
#!/usr/bin/env Rscript

library(rhdf5)

args = commandArgs(trailingOnly=TRUE)
indir <- args[1]
outdir <- args[2]
```

```
fast5 <- dir(indir, "*.fast5",
             recursive = TRUE,
             full.names = TRUE)

null <- lapply(fast5, function(.x)
               {
                   base <- basename(.x)
                   if (!"Events" %in%  h5ls(.x)$name){
                        file.rename(from = .x, to = file.path(outdir, base))
                   }
               })
```

Use this R scripts to remove fast5 files without a "Events" group.

```
#!/bin/bash

#BSUB -n 8  # minmal numbers of processors required for a parallel job
#BSUB -R rusage[mem=1000] # ask for memory 5G
#BSUB -W 4:00 #limit the job to be finished in 12 hours
#BSUB -J "fastQC[1-2]%50"
#BSUB -q short   # which queue we want to run in
#BSUB -o logs/out.%J.%I.txt # log
#BSUB -e logs/err.%J.%I.txt # error
#BSUB -R "span[hosts=1]" # All hosts on the same chassis"
##BSUB -w "done(33544)"

i=$(($LSB_JOBINDEX- 1))
mkdir -p logs

source ~/miniconda3/etc/profile.d/conda.sh

# activate conda environment with the R base and rhdf5 package installed
conda activate seurat5

single_fast5=(`ls data/001.repacked.single.fast5/barcode0*/ -d`)

outdir=data/no.resquiggle.fast5/${out[$i]}
mkdir -p $outdir

Rscript --vanilla  scripts/filter.resquiggle.fast5.R ${single_fast5[$i]}  $outdir
```

Extracting event-specific statistics from the resquiggled single-read

fast5 files

Save the following R scripts as "extract.5mer.R". To run thescripts, the R base future, future.apply, R.utils, and rhdf5 packages need to be installed.

```
#!/usr/bin/env Rscript
library(future.apply)
library(future)
library(rhdf5)
library(R.utils)

plan(multicore)
```

```r
args = commandArgs(trailingOnly=TRUE)
indir <- args[1]
outdir <- args[2]

fast5s <- dir(indir, ".fast5$", full.names = TRUE)
names(fast5s) <- gsub(".+/", "", fast5s)

extract_5mer <- function(fast5, name) {
    events <- h5dump(fast5)$Analyses$RawGenomeCorrected_000$BaseCalled_template$Events
    events$start <- NULL
    T_pos  <- which(events$base == "T")
    if (length(T_pos) >= 1) {
        T_pos <- T_pos[T_pos > 2 & T_pos < nrow(events) -1]
    }

    if (length(T_pos) >= 1) {
        T_centered_5mer_features <- lapply(T_pos, function(.x){
            events[(.x -2):(.x+2), ]
        })

        T_centered_5mer_features <- do.call("rbind", T_centered_5mer_features)

        # Create a gzipped connection
        fname <- gzfile(file.path(outdir, paste0(name, ".resquiggle.features.txt.gz")), "w")
        write.table(T_centered_5mer_features,
                    file= fname,
                    sep = "\t", quote = FALSE,
                    row.names = FALSE)
    }

}

null <- future_mapply(extract_5mer, fast5s, names(fast5s),
                      SIMPLIFY = FALSE,
                      future.chunk.size = 250)
```
