# Supplementary material for: Training Recurrent Neural Networks for BrdU Detection with Oxford Nanopore Sequencing: Guidance and Lessons Learned
Source: Genes (Basel). 2025 Nov 10;16(11):1356. doi: 10.3390/genes16111356 (PMC12652529; doi:10.3390/genes16111356)
Supplement: Supplementary file 1 [file genes-16-01356-s001.zip › File S2. Quality control reports/Dataset I-5-mer reference standard QC report.html]

ToulligQC: BSPS\_BrdU 


Report for BSPS\_BrdU

Sample ID: Unknow   
Run date: Unknown   
Report date: Thu Oct 02 12:30:39 UTC-04:00 2025

- Run statistics
- Device and software
- Read count histogram
- Distribution of read lengths
- PHRED score distribution
- PHRED score density distribution
- Correlation between read length and PHRED score

## Run statistics ⓘ

| Measure | Value |
| --- | --- |
| Report name | BSPS\_BrdU |
| Experiment group | Unknown |
| Sample ID | Unknow |
| Run ID | Unknow |
| Run date | Unknown |
| Run duration | Unknown |
| Flowcell ID | Unknown |
| Flowcell product code | Unknown |
| Flowcell version | Unknown |
| Kit | Unknown |
| Sequencing kit | Unknown |
| Barcode kits | Unknown |
| Selected speed (bps) | Unknown |
| Sample frequency (Hz) | Unknown |
| Yield | 475.38M |
| Read count | 1,037,284 |
| N50 (bp) | 503 |
| L50 | 672,843 |

## Device and software ⓘ

| Measure | Value |
| --- | --- |
| Device type | Unknown |
| Device ID | Unknown |
| Device hostname | Unknown |
| Device OS | Unknown |
| Distribution version | Unknown |
| MinKNOW version | Unknown |
| Basecaller name | Unknown |
| Basecaller version | Unknown |
| Basecaller analysis | Unknown |
| Basecalling date | Unknown |
| Model file | Unknow |
| Min qscore threshold | Unknown |
| ToulligQC version | 2.7.1 |

|  | All reads | Pass reads | Fail reads |
| --- | --- | --- | --- |
| count | 1,037,284 | 1,028,430 | 8,854 |
| percent | 100.00 | 99.15 | 0.85 |

|  | All reads | Pass reads | Fail reads |
| --- | --- | --- | --- |
| count | 1,037,284 | 1,028,430 | 8,854 |
| mean | 458.30 | 457.22 | 582.66 |
| std | 356.45 | 346.07 | 978.82 |
| min | 5.00 | 71.00 | 5.00 |
| 25% | 326.00 | 326.00 | 525.00 |
| median | 373.00 | 372.00 | 596.00 |
| 75% | 596.00 | 595.00 | 635.00 |
| max | 263,061.00 | 263,061.00 | 70,826.00 |

|  | All reads | Pass reads | Fail reads |
| --- | --- | --- | --- |
| count | 1,037,284 | 1,028,430 | 8,854 |
| mean | 12.70 | 12.73 | 8.57 |
| std | 2.09 | 2.07 | 0.85 |
| min | 1.96 | 9.01 | 1.96 |
| 25% | 11.01 | 11.05 | 8.63 |
| median | 12.65 | 12.68 | 8.82 |
| 75% | 14.16 | 14.18 | 8.93 |
| max | 24.30 | 24.30 | 9.00 |


Produced by ToulligQC (version 2.7.1)
