# Supplementary material for: Training Recurrent Neural Networks for BrdU Detection with Oxford Nanopore Sequencing: Guidance and Lessons Learned
Source: Genes (Basel). 2025 Nov 10;16(11):1356. doi: 10.3390/genes16111356 (PMC12652529; doi:10.3390/genes16111356)
Supplement: Supplementary file 1 [file genes-16-01356-s001.zip › File S2. Quality control reports/Dataset II fully BrdU-substituted primer extension data BTF_G_ONT_1_FAD19641_BrdU_fastqc.html]

BTF\_G\_ONT\_1\_FAD19641\_BrdU.fastq.gz FastQC Report 

FastQC Report

Thu 2 Oct 2025  
BTF\_G\_ONT\_1\_FAD19641\_BrdU.fastq.gz

## Summary

- Basic Statistics
- Per base sequence quality
- Per sequence quality scores
- Per base sequence content
- Per sequence GC content
- Per base N content
- Sequence Length Distribution
- Sequence Duplication Levels
- Overrepresented sequences
- Adapter Content

## Basic Statistics

| Measure | Value |
| --- | --- |
| Filename | BTF\_G\_ONT\_1\_FAD19641\_BrdU.fastq.gz |
| File type | Conventional base calls |
| Encoding | Sanger / Illumina 1.9 |
| Total Sequences | 5868 |
| Sequences flagged as poor quality | 0 |
| Sequence length | 650-15790 |
| %GC | 54 |

## Per base sequence quality

## Per sequence quality scores

## Per base sequence content

## Per sequence GC content

## Per base N content

## Sequence Length Distribution

## Sequence Duplication Levels

## Overrepresented sequences

No overrepresented sequences

## Adapter Content

Produced by FastQC (version 0.11.9)
