# Supplementary material for: Training Recurrent Neural Networks for BrdU Detection with Oxford Nanopore Sequencing: Guidance and Lessons Learned
Source: Genes (Basel). 2025 Nov 10;16(11):1356. doi: 10.3390/genes16111356 (PMC12652529; doi:10.3390/genes16111356)
Supplement: Supplementary file 1 [file genes-16-01356-s001.zip › File S2. Quality control reports/Dataset III human data QC report.html]

ToulligQC: barcode02\_human 


Report for barcode02\_human

Sample ID: Unknow   
Run date: Unknown   
Report date: Thu Oct 02 20:02:05 UTC-04:00 2025

- Run statistics
- Device and software
- Read count histogram
- Distribution of read lengths
- PHRED score distribution
- PHRED score density distribution
- Correlation between read length and PHRED score

## Run statistics ⓘ

| Measure | Value |
| --- | --- |
| Report name | barcode02\_human |
| Experiment group | Unknown |
| Sample ID | Unknow |
| Run ID | Unknow |
| Run date | Unknown |
| Run duration | Unknown |
| Flowcell ID | Unknown |
| Flowcell product code | Unknown |
| Flowcell version | Unknown |
| Kit | Unknown |
| Sequencing kit | Unknown |
| Barcode kits | Unknown |
| Selected speed (bps) | Unknown |
| Sample frequency (Hz) | Unknown |
| Yield | 26.67M |
| Read count | 4,011 |
| N50 (bp) | 11,322 |
| L50 | 3,269 |

## Device and software ⓘ

| Measure | Value |
| --- | --- |
| Device type | Unknown |
| Device ID | Unknown |
| Device hostname | Unknown |
| Device OS | Unknown |
| Distribution version | Unknown |
| MinKNOW version | Unknown |
| Basecaller name | Unknown |
| Basecaller version | Unknown |
| Basecaller analysis | Unknown |
| Basecalling date | Unknown |
| Model file | Unknow |
| Min qscore threshold | Unknown |
| ToulligQC version | 2.7.1 |

|  | All reads | Pass reads | Fail reads |
| --- | --- | --- | --- |
| count | 4,011 | 3,370 | 641 |
| percent | 100.00 | 84.02 | 15.98 |

|  | All reads | Pass reads | Fail reads |
| --- | --- | --- | --- |
| count | 4,011 | 3,370 | 641 |
| mean | 6,649.95 | 6,775.00 | 5,987.29 |
| std | 6,674.45 | 6,727.71 | 6,351.17 |
| min | 72.00 | 72.00 | 86.00 |
| 25% | 1,904.00 | 1,968.00 | 1,405.00 |
| median | 4,502.00 | 4,636.00 | 3,798.00 |
| 75% | 9,280.00 | 9,448.00 | 8,184.00 |
| max | 53,851.00 | 53,851.00 | 36,329.00 |

|  | All reads | Pass reads | Fail reads |
| --- | --- | --- | --- |
| count | 4,011 | 3,370 | 641 |
| mean | 13.16 | 14.11 | 8.14 |
| std | 3.12 | 2.41 | 0.53 |
| min | 5.82 | 9.01 | 5.82 |
| 25% | 10.62 | 12.33 | 7.77 |
| median | 13.76 | 14.40 | 8.17 |
| 75% | 15.67 | 15.97 | 8.57 |
| max | 20.25 | 20.25 | 8.99 |


Produced by ToulligQC (version 2.7.1)
