# Supplementary material for: Training Recurrent Neural Networks for BrdU Detection with Oxford Nanopore Sequencing: Guidance and Lessons Learned
Source: Genes (Basel). 2025 Nov 10;16(11):1356. doi: 10.3390/genes16111356 (PMC12652529; doi:10.3390/genes16111356)
Supplement: Supplementary file 1 [file genes-16-01356-s001.zip › File S2. Quality control reports/Dataset III-BrdU-free mouse data QC report.html]

ToulligQC: barcode01\_mouse 


Report for barcode01\_mouse

Sample ID: Unknow   
Run date: Unknown   
Report date: Thu Oct 02 20:02:05 UTC-04:00 2025

- Run statistics
- Device and software
- Read count histogram
- Distribution of read lengths
- PHRED score distribution
- PHRED score density distribution
- Correlation between read length and PHRED score

## Run statistics ⓘ

| Measure | Value |
| --- | --- |
| Report name | barcode01\_mouse |
| Experiment group | Unknown |
| Sample ID | Unknow |
| Run ID | Unknow |
| Run date | Unknown |
| Run duration | Unknown |
| Flowcell ID | Unknown |
| Flowcell product code | Unknown |
| Flowcell version | Unknown |
| Kit | Unknown |
| Sequencing kit | Unknown |
| Barcode kits | Unknown |
| Selected speed (bps) | Unknown |
| Sample frequency (Hz) | Unknown |
| Yield | 36.48M |
| Read count | 4,012 |
| N50 (bp) | 16,245 |
| L50 | 3,278 |

## Device and software ⓘ

| Measure | Value |
| --- | --- |
| Device type | Unknown |
| Device ID | Unknown |
| Device hostname | Unknown |
| Device OS | Unknown |
| Distribution version | Unknown |
| MinKNOW version | Unknown |
| Basecaller name | Unknown |
| Basecaller version | Unknown |
| Basecaller analysis | Unknown |
| Basecalling date | Unknown |
| Model file | Unknow |
| Min qscore threshold | Unknown |
| ToulligQC version | 2.7.1 |

|  | All reads | Pass reads | Fail reads |
| --- | --- | --- | --- |
| count | 4,012 | 3,987 | 25 |
| percent | 100.00 | 99.38 | 0.62 |

|  | All reads | Pass reads | Fail reads |
| --- | --- | --- | --- |
| count | 4,012 | 3,987 | 25 |
| mean | 9,092.23 | 9,133.82 | 2,460.32 |
| std | 8,972.85 | 8,974.33 | 5,776.10 |
| min | 74.00 | 84.00 | 74.00 |
| 25% | 2,158.00 | 2,210.00 | 133.00 |
| median | 6,115.50 | 6,156.00 | 210.00 |
| 75% | 13,345.25 | 13,403.50 | 1,426.00 |
| max | 50,241.00 | 50,241.00 | 28,113.00 |

|  | All reads | Pass reads | Fail reads |
| --- | --- | --- | --- |
| count | 4,012 | 3,987 | 25 |
| mean | 14.02 | 14.06 | 8.37 |
| std | 2.09 | 2.04 | 0.94 |
| min | 4.77 | 9.01 | 4.77 |
| 25% | 12.54 | 12.57 | 8.28 |
| median | 14.26 | 14.28 | 8.78 |
| 75% | 15.59 | 15.60 | 8.90 |
| max | 19.42 | 19.42 | 9.00 |


Produced by ToulligQC (version 2.7.1)
