# Supplementary material for: Training Recurrent Neural Networks for BrdU Detection with Oxford Nanopore Sequencing: Guidance and Lessons Learned
Source: Genes (Basel). 2025 Nov 10;16(11):1356. doi: 10.3390/genes16111356 (PMC12652529; doi:10.3390/genes16111356)
Supplement: Supplementary file 1 [file genes-16-01356-s001.zip › File S2. Quality control reports/Dataset IV BO_testing_1 BrdU-free yeast data QC report.html]

ToulligQC: BO 


Report for BO

Sample ID: Unknow   
Run date: Unknown   
Report date: Thu Oct 02 17:05:48 UTC-04:00 2025

- Run statistics
- Device and software
- Read count histogram
- Distribution of read lengths
- PHRED score distribution
- PHRED score density distribution
- Correlation between read length and PHRED score

## Run statistics ⓘ

| Measure | Value |
| --- | --- |
| Report name | BO |
| Experiment group | Unknown |
| Sample ID | Unknow |
| Run ID | Unknow |
| Run date | Unknown |
| Run duration | Unknown |
| Flowcell ID | Unknown |
| Flowcell product code | Unknown |
| Flowcell version | Unknown |
| Kit | Unknown |
| Sequencing kit | Unknown |
| Barcode kits | Unknown |
| Selected speed (bps) | Unknown |
| Sample frequency (Hz) | Unknown |
| Yield | 69.32M |
| Read count | 4,001 |
| N50 (bp) | 31,940 |
| L50 | 3,241 |

## Device and software ⓘ

| Measure | Value |
| --- | --- |
| Device type | Unknown |
| Device ID | Unknown |
| Device hostname | Unknown |
| Device OS | Unknown |
| Distribution version | Unknown |
| MinKNOW version | Unknown |
| Basecaller name | Unknown |
| Basecaller version | Unknown |
| Basecaller analysis | Unknown |
| Basecalling date | Unknown |
| Model file | Unknow |
| Min qscore threshold | Unknown |
| ToulligQC version | 2.7.1 |

|  | All reads | Pass reads | Fail reads |
| --- | --- | --- | --- |
| count | 4,001 | 3,775 | 226 |
| percent | 100.00 | 94.35 | 5.65 |

|  | All reads | Pass reads | Fail reads |
| --- | --- | --- | --- |
| count | 4,001 | 3,775 | 226 |
| mean | 17,325.22 | 17,502.15 | 14,369.89 |
| std | 16,936.12 | 16,874.59 | 17,711.75 |
| min | 186.00 | 186.00 | 287.00 |
| 25% | 3,121.00 | 3,177.50 | 2,368.00 |
| median | 12,541.00 | 12,740.00 | 6,668.50 |
| 75% | 27,161.00 | 27,397.00 | 21,339.25 |
| max | 113,543.00 | 113,543.00 | 104,475.00 |

|  | All reads | Pass reads | Fail reads |
| --- | --- | --- | --- |
| count | 4,001 | 3,775 | 226 |
| mean | 14.99 | 15.44 | 7.46 |
| std | 2.94 | 2.35 | 0.84 |
| min | 4.78 | 9.03 | 4.78 |
| 25% | 13.47 | 13.93 | 6.92 |
| median | 15.75 | 15.92 | 7.28 |
| 75% | 17.09 | 17.18 | 8.11 |
| max | 25.16 | 25.16 | 8.99 |


Produced by ToulligQC (version 2.7.1)
