# Supplementary material for: Training Recurrent Neural Networks for BrdU Detection with Oxford Nanopore Sequencing: Guidance and Lessons Learned
Source: Genes (Basel). 2025 Nov 10;16(11):1356. doi: 10.3390/genes16111356 (PMC12652529; doi:10.3390/genes16111356)
Supplement: Supplementary file 1 [file genes-16-01356-s001.zip › File S2. Quality control reports/Dataset IV BY_testing_1 80.3% BrdU substituion yeast data QC report.html]

ToulligQC: BO 


Report for BO

Sample ID: Unknow   
Run date: Unknown   
Report date: Thu Oct 02 17:05:48 UTC-04:00 2025

- Run statistics
- Device and software
- Read count histogram
- Distribution of read lengths
- PHRED score distribution
- PHRED score density distribution
- Correlation between read length and PHRED score

## Run statistics ⓘ

| Measure | Value |
| --- | --- |
| Report name | BO |
| Experiment group | Unknown |
| Sample ID | Unknow |
| Run ID | Unknow |
| Run date | Unknown |
| Run duration | Unknown |
| Flowcell ID | Unknown |
| Flowcell product code | Unknown |
| Flowcell version | Unknown |
| Kit | Unknown |
| Sequencing kit | Unknown |
| Barcode kits | Unknown |
| Selected speed (bps) | Unknown |
| Sample frequency (Hz) | Unknown |
| Yield | 37.60M |
| Read count | 4,001 |
| N50 (bp) | 22,074 |
| L50 | 3,462 |

## Device and software ⓘ

| Measure | Value |
| --- | --- |
| Device type | Unknown |
| Device ID | Unknown |
| Device hostname | Unknown |
| Device OS | Unknown |
| Distribution version | Unknown |
| MinKNOW version | Unknown |
| Basecaller name | Unknown |
| Basecaller version | Unknown |
| Basecaller analysis | Unknown |
| Basecalling date | Unknown |
| Model file | Unknow |
| Min qscore threshold | Unknown |
| ToulligQC version | 2.7.1 |

|  | All reads | Pass reads | Fail reads |
| --- | --- | --- | --- |
| count | 4,001 | 1,151 | 2,850 |
| percent | 100.00 | 28.77 | 71.23 |

|  | All reads | Pass reads | Fail reads |
| --- | --- | --- | --- |
| count | 4,001 | 1,151 | 2,850 |
| mean | 9,397.25 | 8,008.57 | 9,958.08 |
| std | 12,060.12 | 12,094.27 | 12,002.96 |
| min | 184.00 | 198.00 | 184.00 |
| 25% | 1,400.00 | 616.00 | 1,881.25 |
| median | 4,130.00 | 2,280.00 | 4,892.00 |
| 75% | 12,917.00 | 10,184.00 | 13,856.00 |
| max | 102,113.00 | 100,918.00 | 102,113.00 |

|  | All reads | Pass reads | Fail reads |
| --- | --- | --- | --- |
| count | 4,001 | 1,151 | 2,850 |
| mean | 9.07 | 14.24 | 6.98 |
| std | 3.66 | 2.84 | 0.62 |
| min | 4.40 | 9.01 | 4.40 |
| 25% | 6.73 | 11.93 | 6.62 |
| median | 7.14 | 14.56 | 6.88 |
| 75% | 10.21 | 16.53 | 7.25 |
| max | 22.32 | 22.32 | 8.98 |


Produced by ToulligQC (version 2.7.1)
