# Supplementary figures and images for: Training Recurrent Neural Networks for BrdU Detection with Oxford Nanopore Sequencing: Guidance and Lessons Learned
Source: Genes (Basel). 2025 Nov 10;16(11):1356. doi: 10.3390/genes16111356 (PMC12652529; doi:10.3390/genes16111356)

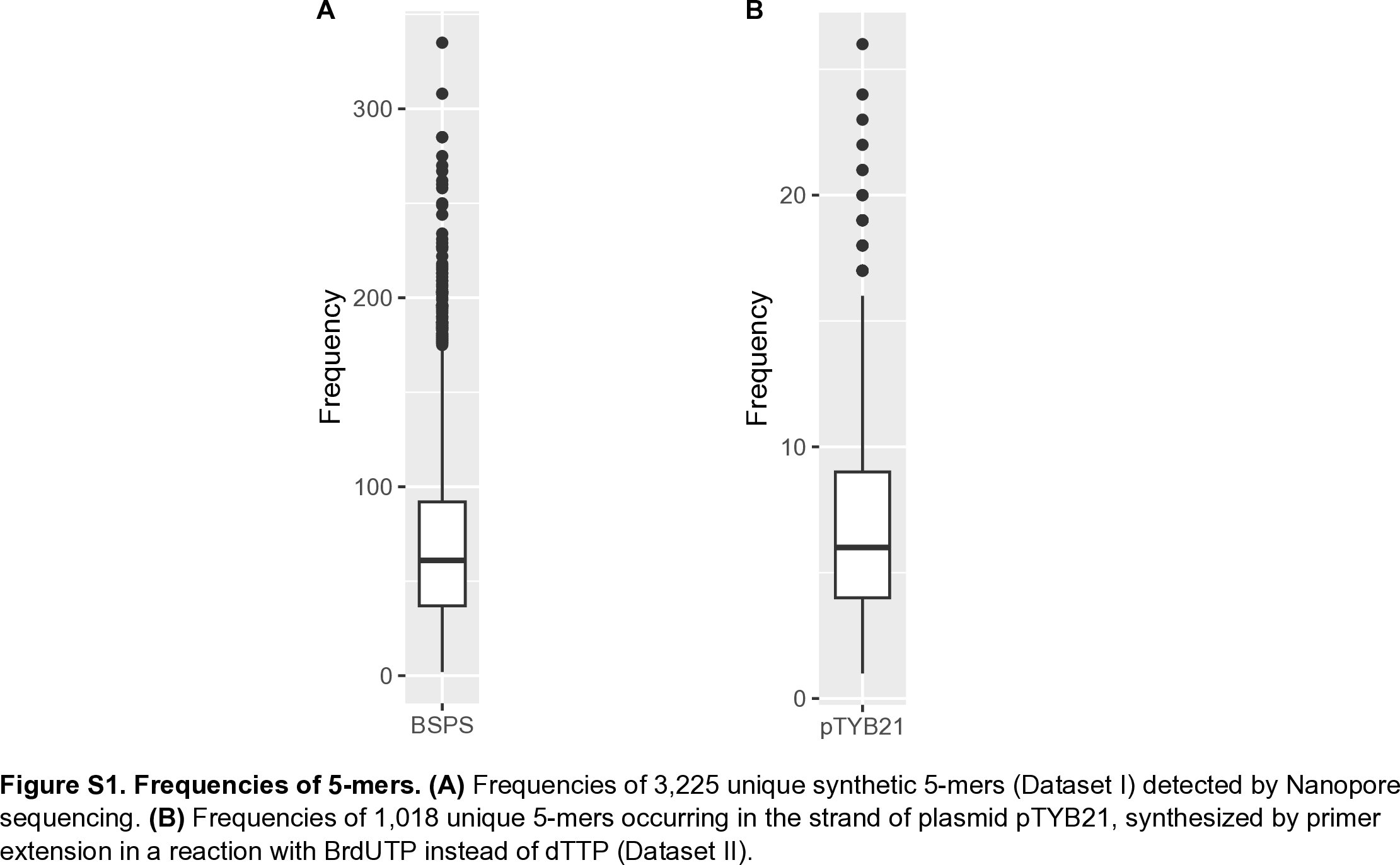

Supplement: Supplementary file 1 [file genes-16-01356-s001.zip › Figure S1.tif]
